# Formation of Complex and Unstable Chromosomal Translocations in Yeast

**DOI:** 10.1371/journal.pone.0012007

**Published:** 2010-08-09

**Authors:** Kristina H. Schmidt, Emilie Viebranz, Lillian Doerfler, Christina Lester, Aaron Rubenstein

**Affiliations:** Department of Cell Biology, Microbiology and Molecular Biology, University of South Florida, Tampa, Florida, United States of America; University of Minnesota, United States of America

## Abstract

Genome instability, associated with chromosome breakage syndromes and most human cancers, is still poorly understood. In the yeast *Saccharomyces cerevisiae*, numerous genes with roles in the preservation of genome integrity have been identified. DNA-damage-checkpoint-deficient yeast cells that lack Sgs1, a RecQ-like DNA helicase related to the human Bloom's-syndrome-associated helicase BLM, show an increased rate of genome instability, and we have previously shown that they accumulate recurring chromosomal translocations between three similar genes, *CAN1*, *LYP1* and *ALP1*. Here, the chromosomal location, copy number and sequence similarity of the translocation targets *ALP1* and *LYP1* were altered to gain insight into the formation of complex translocations. Among 844 clones with chromosomal rearrangements, 93 with various types of simple and complex translocations involving *CAN1*, *LYP1* and *ALP1* were identified. Breakpoint sequencing and mapping showed that the formation of complex translocation types is strictly dependent on the location of the initiating DNA break and revealed that complex translocations arise via a combination of interchromosomal translocation and template-switching, as well as from unstable dicentric intermediates. Template-switching occurred between sequences on the same chromosome, but was inhibited if the genes were transferred to different chromosomes. Unstable dicentric translocations continuously gave rise to clones with multiple translocations in various combinations, reminiscent of intratumor heterogeneity in human cancers. Base substitutions and evidence of DNA slippage near rearrangement breakpoints revealed that translocation formation can be accompanied by point mutations, and their presence in different translocation types within the same clone provides evidence that some of the different translocation types are derived from each other rather than being formed *de novo*. These findings provide insight into eukaryotic genome instability, especially the formation of translocations and the sources of intraclonal heterogeneity, both of which are often associated with human cancers.

## Introduction

Structural changes to chromosomes, such as translocations, terminal fusions, insertions, inversions or deletions, are often detrimental to normal cell proliferation and are commonly associated with cancers, accelerated aging and genomic disorders [1], [2], [3]. They are thought to result from non-homologous endjoining (NHEJ) of double-strand breaks (DSBs) or from erroneous homologous recombination (HR) between dispersed, nonallelic repeats (NAHR). HR events are initiated by 3′ end invasion of identical duplex DNA, normally on a homologous chromosome or a sister chromatid or, accidentally, nonallelic sequences. Break-induced replication (BIR) has been invoked as an HR mechanism for the repair of one-sided DSBs that may arise when a replication fork collapses at a nick in the template or when telomeres erode [4], [5], [6], [7]. BIR is a Rad52-dependent mechanism and requires long homology for successful strand invasion; however, BIR requiring only microhomology has recently been proposed as a mechanism for generating copy number variation in the human genome [8]. In addition, recent evidence from yeast suggests that broken replication forks may also be substrates for an HR-protein independent, replication-based template-switching mechanism that is mediated by microhomology or microsatellites [9]. Despite these recent advances, genetic and mechanistic understanding of the causes of genome instability in model organisms as well as in human genome instability syndromes and cancer is still lacking. With the identification of numerous genes and gene networks that are required for the maintenance of genome stability, including DNA damage checkpoints, DNA repair factors, proteins for processing of recombination substrates, as well as components of chromatin assembly factors, the budding yeast *Saccharomyces cerevisiae* has emerged as a model organism for the study of genome instability [10], [11], [12], [13], [14], [15], [16], [17], [18], [19]. Members of the RecQ family of DNA helicases have been recognized as important regulators of genome integrity from bacteria to humans (reviewed in [20]). Yeast cells lacking the RecQ-like helicase Sgs1 accumulate gross-chromosomal rearrangements (GCRs), exhibit elevated levels of mitotic recombination, have a reduced lifespan and are sensitive to chemicals that alkylate DNA or slow replication forks [14], [20], [21], [22], [23], [24]. *In vitro*, Sgs1 is capable of unwinding various DNA substrates, but prefers Holliday junctions, consistent with its proposed role in recombination [25]. Sgs1 has also been shown to facilitate formation of the 3′ overhang during the processing of DSBs in preparation for strand invasion [26]. In humans, lack of function of the RecQ-like DNA helicases BLM, WRN and RECQL4 is associated with Bloom's, Werner and Rothmund-Thompson syndromes, respectively, which are characterized by chromosome abnormalities, increased cancer susceptibility and/or signs of premature aging [27], [28], [29]. Not unlike yeast cells lacking Sgs1, cells from Bloom's syndrome patients exhibit aberrant and/or elevated levels of genetic exchange and chromosome instability. The most striking characteristics of cells from Bloom's syndrome patients include elevated rates of sister-chromatid exchange, chromatid gaps, micronuclei and quadriradial structures [30], [31].

In an ongoing effort to elucidate genetic and mechanistic determinants of chromosome instability in yeast, we previously identified various types of complex, recurring translocations between three homeologous genes in yeast cells that lack Sgs1 and the DNA-damage sensor Mec3 [18]. A candidate screen revealed that deletion of other checkpoint components (Tel1, Rfc5, Rad24) or deletion of chromatin assembly factors (Cac1, Asf1) also made *sgs1Δ* mutants susceptible to these recurring translocations [19]. We determined that these translocations originate in the *CAN1* gene on chromosome V and target short stretches of identical sequences in the related genes *LYP1* and/or *ALP1* on chromosome XIV, which share 60–65% sequence identity with each other and with *CAN1*. Using the highly susceptible *sgs1Δ mec3Δ* mutant as a source for translocations, the goal of the present study was to gain insight into how the various simple and complex translocations between *CAN1*, *LYP1* and *ALP1*, and possibly chromosomal translocations in general, are formed. For this purpose, we manipulated the location, copy number and level of sequence similarity of the translocation targets *ALP1* and *LYP1* and determined the effect of these changes on the accumulation, structure and stability of the translocation chromosomes. We find that complex, multipartite translocations only form if sequences of sufficient similarity are available on the same chromosome for template-switching, whereas translocation formation involving two successive interchromosomal rearrangements were not observed. Rather than giving rise to inviable cells, dicentric chromosomes provide a continuous source for new viable translocations and show signs of ongoing instability that leads to chromosome end erosion. Point mutations and DNA slippage events that accompany some rearrangements give further insight into the origin of stable translocations.

## Materials and Methods

### Yeast Strains, Plasmids and Media

All strains used in this study are derived from *Saccharomyces cerevisiae* strain S288C and are listed in Table 1. For GCR rate measurements, desired gene deletions were introduced into KHSY802 (*MATa*, *ura3-52*, *trp1Δ63*, *his3Δ200*, *leu2Δ1*, *lys2Bgl*, *hom3-10*, *ade2Δ1*, *ade8*, *hxt13::URA3*), RDKY5027 (*MATα*, *ura3-52*, *trp1Δ63*, *his3Δ200*, *leu2Δ1*, *lys2Bgl*, *hom3-10*, *ade2Δ1*, *ade8*, *hxt13::URA3*) by HR-mediated integration of PCR products by the LiAc method [32]. All haploid strains for GCR rate measurements were obtained by sporulating diploids heterozygous for the desired mutations. Spores were genotyped on selective media or by PCR. Media for propagating strains have been previously described [33]. *ALP1* on chromosome XIV was deleted by inserting the *loxP-kanMX6-loxP* cassette from pUG6 (gift from S. Brill, Rutgers University) at *ALP1*, followed by Cre-recombinase-mediated removal of the *kanMX6* cassette. To insert *ALP1* into chromosome II, the *ALP1* ORF was first inserted into pCR4 (Invitrogen) and a *kanMX6* cassette was inserted downstream of *ALP1* into the *Spe*I site of pCR4, yielding plasmid pKHS332. The *ALP1*.*kanMX6* cassette from pKHS332 was then amplified by PCR and inserted into chromosome II downstream of *HIS7* between nucleotides 714705 and 714707 in a yeast strain that had *ALP1* on chromosome XIV deleted (*ALP1::loxP*) to improve the targeting efficiency to chromosome II. For site-directed mutagenesis, *LYP1* was inserted into pCR2.1 (Invitrogen) and base substitutions A879T, C885T, G902A, A906G, C927T, C933T, C981A (*LYP1*-MUTABCDEF) were introduced using the QuickChange protocol (Stratagene), to generate plasmid pKHS318. A *loxP-kanMX-loxP* cassette was inserted into the *PmeI* site of pKHS318 and, together with *lyp1*-*MUTABCDEF*, used to replace the chromosomal *LYP1*. The *kanMX6* cassette was excised from the chromosomal integration by transient Cre-recombinase expression. Unless notes otherwise, the *CAN1* gene is in its wildtype location on chromosome V and a *URA3* cassette was used to replace the *HXT13* gene on chromosome V [10], [34]. In the strain designated HR-wt, the *LYP1* and *ALP1* genes are at their wildtype loci on chromosome XIV (KHSY1530). HR-1 is identical to HR-wt except that a second copy of *ALP1* was inserted into chromosome II as described above (KHSY2147). In HR-2 *LYP1* is in the wildtype location whereas *ALP1* on chromosome XIV was deleted and a copy of *ALP1* was inserted into chromosome II (KHSY2612). In HR-3, *ALP1* on chromosome XIV was deleted and no other copy of *ALP1* exists in this strain (KHSY2098). In HR-4 *ALP1* is in its wildtype location whereas *LYP1* was replaced with the mutant *LYP1* allele containing A879T, C885T, G902A, A906G, C927T, C933T, C981A base substitutions (KHSY3114).

**Table 1:** *Saccharomyces cerevisiae* strains used in this study.

Strain number	Genotype
KHSY1530	*MATa*, *ura3-52*, *trp1Δ63*, *his3Δ200*, *leu2Δ1*, *lys2ΔBgl*, *hom3-10*, *ade2Δ1*, *ade8*, *hxt13::URA3*, *sgs1::TRP1*, *mec3::HIS3*
KHSY2098	*MATa*, *ura3-52*, *trp1Δ63*, *his3Δ200*, *leu2Δ1*, *lys2ΔBgl*, *hom3-10*, *ade2Δ1*, *ade8*, *hxt13::URA3*, *sgs1::TRP1*, *mec3::HIS3*, *alp1::loxP*
KHSY2147	*MATa*, *ura3-52*, *trp1Δ63*, *his3Δ200*, *leu2Δ1*, *lys2ΔBgl*, *hom3-10*, *ade2Δ1*, *ade8*, *hxt13::URA3*, *sgs1::TRP1*, *mec3::HIS3*, *ALP1.kanMX6(chrIIins714705)*
KHSY2612	*MATa*, *ura3-52*, *trp1Δ63*, *his3Δ200*, *leu2Δ1*, *lys2ΔBgl*, *hom3-10*, *ade2Δ1*, *ade8*, *hxt13::URA3*, *sgs1::TRP1*, *mec3::HIS3*, *alp1::loxP*, *ALP1.kanMX6(chrIIins714705)*
KHSY3114	*MATa*, *ura3-52*, *trp1D63*, *his3D200*, *leu2D1*, *lys2DBgl*, *hom3-10*, *ade2D1*, *ade8*, *hxt13::URA3*, *sgs1::TRP1*, *mec3::HIS3*, *lyp1-MUTABCDEF.loxP*

### Identification of Translocations Involving *CAN1*, *LYP1* and *ALP1*

Clones with spontaneous gross-chromosomal rearrangements (GCRs) that originate in a 12-kb nonessential region of chromosome V, which contains *CAN1*, were obtained exactly as previously described [34]. To identify GCR clones with translocations involving *CAN1* and *LYP1* and/or *ALP1*, GCR clones were screened by PCR. A primer pair that anneals to the 5′ end of *CAN1* and to the 3′end of *LYP1* was used to amplify *C/L* translocations and a primer pair that anneals to the 5′end of *CAN1* and the 3′end of *ALP1* was used to amplify *C/A* and *C/L/A* translocations. PCR products were sequenced and analyzed by BLAST and Sequencher (GeneCodes) to distinguish between *C/A* and *C/L/A* translocations and to identify fusion sites. Translocations terminating in *ALP1* on chromosome XIV were distinguished from those terminating in *ALP1* on chromosome II by PCR using a primer pair that anneals to the 5′ end of *CAN1* and downstream of *ALP1* ORF on chromosome XIV, or a primer pair that anneals to the 5′end of *CAN1* and within the *kanMX6* cassette linked to the *ALP1* ORF insertion on chromosome II, respectively.

### Comparative Genome Hybridization (CGH)

Genomic DNA was extracted from a YPD culture inoculated with a single colony of the GCR clone. Proteins were removed by three rounds of phenol-chloroform-isoamylalcohol extraction. Ten micrograms of genomic DNA at a concentration of 250 ng/µl were used per array. The parental strain RDKY3615 with an intact chromosome V was used as the reference genomic DNA. Hybridization, array scanning and data extraction are performed by NimbleGen Systems, Inc. The CGH array used for this analysis covers the *S. cerevisiae* genome using 45–85mer isothermal probes with a median probe spacing of 12 bp.

## Results

### Dependency of Complex Translocations on Intrachromosomal Template-Switching

**Figure 1:**
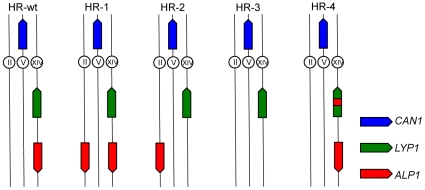
Modification of location, copy number and sequence similarity of *ALP1* and *LYP1*. In unmodified cells (HR-wt), *CAN1* (blue) is on chromosome V and *LYP1* (green) and *ALP1* (red) are in opposite orientations on the same arm of chromosome XIV. Two copies of *ALP1* were present on chromosome XIV and II in HR-1, *ALP1* was moved from chromosome XIV to II in HR-2, *ALP1* was deleted in HR-3 and sequence similarity between HER-II of *ALP1* and *LYP1* was increased from 96% to 100% in HR-4.

Previously we showed that cells lacking Sgs1 and the DNA damage sensor Mec3 are particularly susceptible to translocations between *CAN1* and *ALP1* (*C/A*), *CAN1* and *LYP1* (*C/L*), or even all three related genes (*C/L/A*) [19]. Unexpectedly, the more complex tripartite *C/L/A* translocations arise as frequently as the simple *C/A* translocations, leading us to hypothesize that intrachromosomal rearrangements between the *LYP1* and *ALP1* genes, which are located on the same arm of chromosome XIV, may promote tripartite translocation formation. Here, to elucidate the formation of these tripartite translocations, the *ALP1* and *LYP1* loci were modified in an *sgs1Δ mec3Δ* mutant and the effect of these manipulations on the rate and type of translocations as well as on gene fusion site selection was determined. In addition to the yeast strain with *CAN1*, *LYP1* and *ALP1* in their wildtype locations (HR-wt), four new strains were constructed (Figure 1). The first strain, HR-1, contains a second copy of *ALP1* on chromosome II in the same orientation and at a distance from the telomere similar to that of *ALP1* on chromosome XIV. In this strain, *ALP1* on chromosome XIV competes with *ALP1* on chromosome II as a translocation target for *LYP1*. While *ALP1* on chromosome XIV can be utilized for *intra*chromosomal rearrangements with *LYP1*, *ALP1* on chromosome II can be utilized for *inter*chromosomal rearrangements with *LYP1*. Thus, in theory, the complex *C/L/A* translocations in HR-1 can arise either by rearrangement between chromosomes V and XIV, or by rearrangement between three different chromosomes. The standard GCR assay, which selects for clones that had suffered a spontaneous DNA break within a 12 kb region on chromosome V that also includes the *CAN1* gene [34], was used to collect 423 clones from HR-1 with various chromosome V rearrangements, which may include *de novo* telomere additions, insertions, inversions, large interstitial deletions as well as translocations. Among those 423 clones, 65 clones in which a broken *CAN1* gene on chromosome V had rearranged with *LYP1* and/or *ALP1*, were identified (Table 2). Translocations targeting *ALP1* on chromosome II were distinguished from those targeting *ALP1* on chromosome XIV using a PCR primer that anneals downstream of *ALP1* on chromosome II, but not on chromosome XIV. The frequency of all *CAN1/LYP1/ALP1* translocations in HR-1 (15%, 65/423) was similar to that of the wildtype strain (13%, 20/150) and *C/A* and *C/L/A* translocations terminating in chromosome XIV formed readily. However, no *C/L/A* translocations terminating in *ALP1* on chromosome II were found. This lack of *C/L/A* translocations with chromosome II is not due to unavailability of *ALP1* on chromosome II as a suitable translocation target since *C/A* translocations involving *ALP1* on chromosome II were frequent (45/65). Instead, it demonstrates that *inter*chromosomal rearrangements between *LYP1* and *ALP1* do not form. To verify this finding, a second strain was constructed, HR-2, in which *ALP1* on chromosome II was kept, but the second copy of *ALP1* on chromosome XIV was deleted so that there was no competition between two *ALP1* copies (Figure 1, HR-2). Indeed, when we screened chromosome V rearrangements in HR-2, no *C/L/A* translocations were observed (0/166). This absence of *C/L/A* translocations with chromosome II suggests that tripartite translocation formation depends on an intrachromosomal, secondary rearrangement, such as template-switching between similar DNA sequences. Thus, we reasoned that facilitating this intrachromosomal rearrangement between *LYP1* and *ALP1* by increasing sequence identity between *LYP1* and *ALP1* should lead to an increase in the formation of *C/L/A* translocations. To test this possibility, seven single nucleotides in *LYP1* were changed to perfectly match *ALP1*, extending the length of identical sequences between the two genes, which range from 5–41 bp in the wildtype genes, to a single region of 173 identical base pairs in strain HR-4 (Figure 1). Surprisingly, neither the overall GCR rate (1.1×10^−7^) nor the rate of *CAN1*/*LYP1*/*ALP1* translocations (9.4×10^−9^) increased in HR-4 when compared to HR-wt (GCR rate: 1.3×10^−7^, *CAN1*/*LYP1*/*ALP1* translocation rate: 1.7×10^−8^). That increasing the similarity of *LYP1* and *ALP1* did not affect translocation rates or translocation types suggests that the conversion of dicentric *C/L* translocations into monocentric *C/L/A* translocations may not be the rate-limiting step in translocation formation. Instead, the success of the initial translocation between *CAN1* and *LYP1* may determine the translocation rate, and experiments are currently underway to test this possibility. Finally, we wanted to assess if *C/L* translocations were so rare because they were promptly converted into *C/L/A* translocations or because cells harboring dicentrics could not grow into colonies. For this purpose, *ALP1* was deleted from the genome (Figure 1, HR-3) and GCR clones were screened for *C/L* translocations. That none were found suggests that most translocation chromosomes with *C/L* fusions do not survive unless *ALP1* is available for a secondary rearrangement that converts the dicentric into a monocentric chromosome.

**Table 2:** Effect of changes in translocation target location, copy number and sequence identity on structure of spontaneous translocations involving the *CAN1*, *LYP1* and/or *ALP1* loci.

Translocation Type^i^	HR-wt^ii^	HR-1	HR-2	HR-3	HR-4
*C/A^chrXIV^*	7	6	n.a.	n.a.	1
*C/A^chrII^*	n.a.	46	1	n.a.	n.a.
*C/L*	3	0	1	0	0
*C/L/A^chrXIV^*	7	12	n.a.	n.a.	4
*C/L/A^chrII^*	n.a.	0	0	n.a.	n.a.
Otheriii	3	1	1	0	0
Translocation Frequency	13% (20/150)	15% (65/423)	1.8% (3/166)	0% (0/45)	8% (5/60)

i Translocations types *C/A^chrXIV^* and *C/A^chrII^* refer to *C/A* translocations terminating in *ALP1* on chromosome XIV or II, respectively. Translocation types *C/L/A^chrXIV^* and *C/L/A^chrII^* refer to *C/L/A* translocations terminating in *ALP1* on chromosome XIV or II, respectively.

ii HR-wt, HR-1, HR-2, HR-3 and HR-4 refer to strains KHSY1530, KHSY2147, KHSY2612, KHSY2098, and KHSY3114, respectively. n.a., not available.

iii ‘Other’ refers to clones with translocation types other than one of three major types of *C/A*, *C/L/A* and *C/L* translocations, including clones with multiple translocation type.

### Breakpoint Site Selection does not Depend on Chromosomal Target Location but Shows a Positive Correlation with 5′ Homology Length

**Figure 2:**
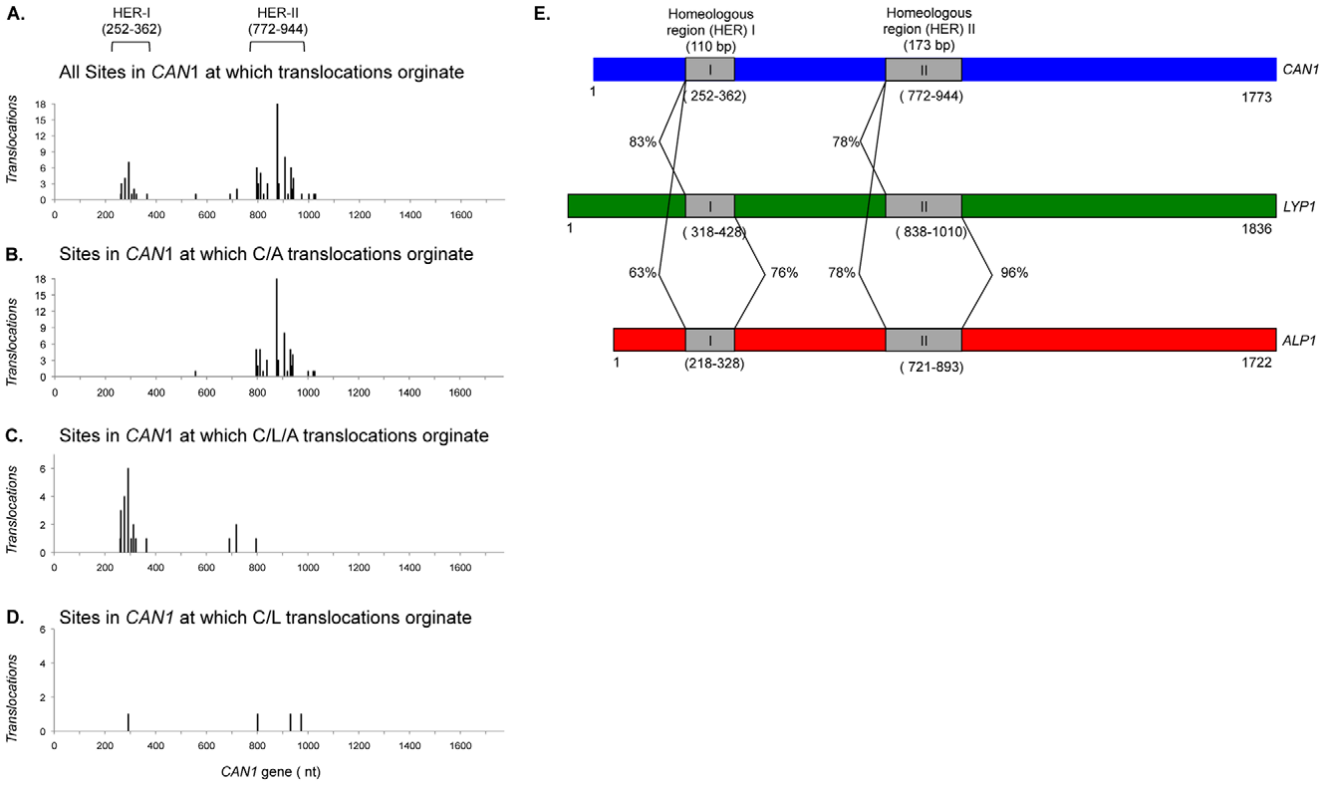
Breakpoint locations in *CAN1*. *C/A*, *C/L/A* and *C/L* translocations were sequenced and the last nucleotide of the *CAN1* gene was mapped to the 1773-bp *CAN1* gene. (**A**) The vast majority of breakpoints fall within Homeologous region I (HER-I) or Homeologous region II (HER-II), whereas further analysis reveals that (**B**) *C/A* translocations originate from HER-II, and (**C**) *C/L/A* translocations originate from HER-I of *CAN1*. (**D**) *CAN1* breakpoints of dicentric *C/L* translocations can fall into HER-I or HER-II depending on availability of *ALP1* for a secondary rearrangement. (**E**) Location and shared sequence identity of the breakpoint clusters HER-I and HER-II in *CAN1*, *LYP1* and *ALP1*. HER-I spans 110 bp and shows greater similarity between *CAN1* and *LYP1* than *ALP1*. HER-II spans 173 bp and shows 96% sequence identity between *LYP1* and *ALP1* whereas *CAN1* shares only 78% sequence identity with *LYP1* and *ALP1* in that region. HER-I and HER-II are the two largest regions of greatest sequence identity present in these three genes. Over the entire ORF, *CAN1*, *LYP1* and *ALP1* share 60–65% of their sequence. Sequences are shown to scale and are aligned at the HER-I and HER-II regions.

The *CAN1*, *LYP1* and *ALP1* genes share 60–65% overall sequence identity, and we previously reported that rearrangements between *LYP1* and *ALP1* more often occurred in longer stretches of identical sequences than expected by chance, consistent with a homology-driven, Rad52-dependent translocation mechanism [19]. In order to determine how the modifications of *ALP1* and *LYP1* had affected breakpoint selection, sites in *CAN1* and *LYP1* where translocations originate (donor sites) and sites in *LYP1* and *ALP1* at which translocations are aimed (target sites) were amplified and sequenced in all 93 translocation isolated from the unmodified and the modified strains. In this study, the term ‘breakpoint’ is used to describe the sites within the *CAN1*, *LYP1* and *ALP1* genes at which the nucleotide sequence of one gene is fused to the nucleotide sequence of another gene; thus the term ‘breakpoint’ most likely refers to the sites where recombination events were resolved rather than sites at which the initiating DNA lesion occurred. We first identified all sites in *CAN1* at which translocations originate and found that 89% of them cluster within two regions, which together span only 283 bp of the 1773-bp *CAN1* gene (Figure 2 A). The first cluster spans 110 bp and is hereafter referred to as homeologous region I, HER-I. While *CAN1* and *LYP1* share 83% of HER-I sequence, only 63% similarity exists with *ALP1* (Figure 2 E). Moreover, the *CAN1*-*LYP1* alignment also shows fewer gaps and longer continuous stretches of matching sequences, suggesting that the 5′ end of *LYP1* may be the preferred target for *CAN1* invasion (Figure S1). The second breakpoint cluster, HER-II, was noticed in our previous study. It spans 173 bp, with *CAN1* sharing 78% of sequence with both *LYP1* and *ALP1*, but with *LYP1* and *ALP1* sharing 96% with each other (Figure 2 E). When sorted by translocation type, it emerged that HER-I facilitates *C/L/A* translocations (Figure 2 C) and HER-II facilitates *C/A* (Figure 2 B) and *C/L* translocations (Figure 2 D). Not a single one of the 47 *C/A* translocation originated in HER-I, suggesting that the 63% sequence similarity between the HER-I regions of *CAN1* and *ALP1* is not sufficient for an interchromosomal translocation, whereas 83% identity between the HER-I regions of *CAN1* and *LYP1* appears sufficient. Taken together, this finding demonstrates that a 110-bp region of 83% sequence identity and with homology blocks not exceeding 14 bp in length is sufficient for Rad52-dependent break-induced replication in yeast cells lacking Sgs1 and Mec3, but not in wildtype cells or in the single mutants, in which these translocations are not observed.

**Figure 3:**
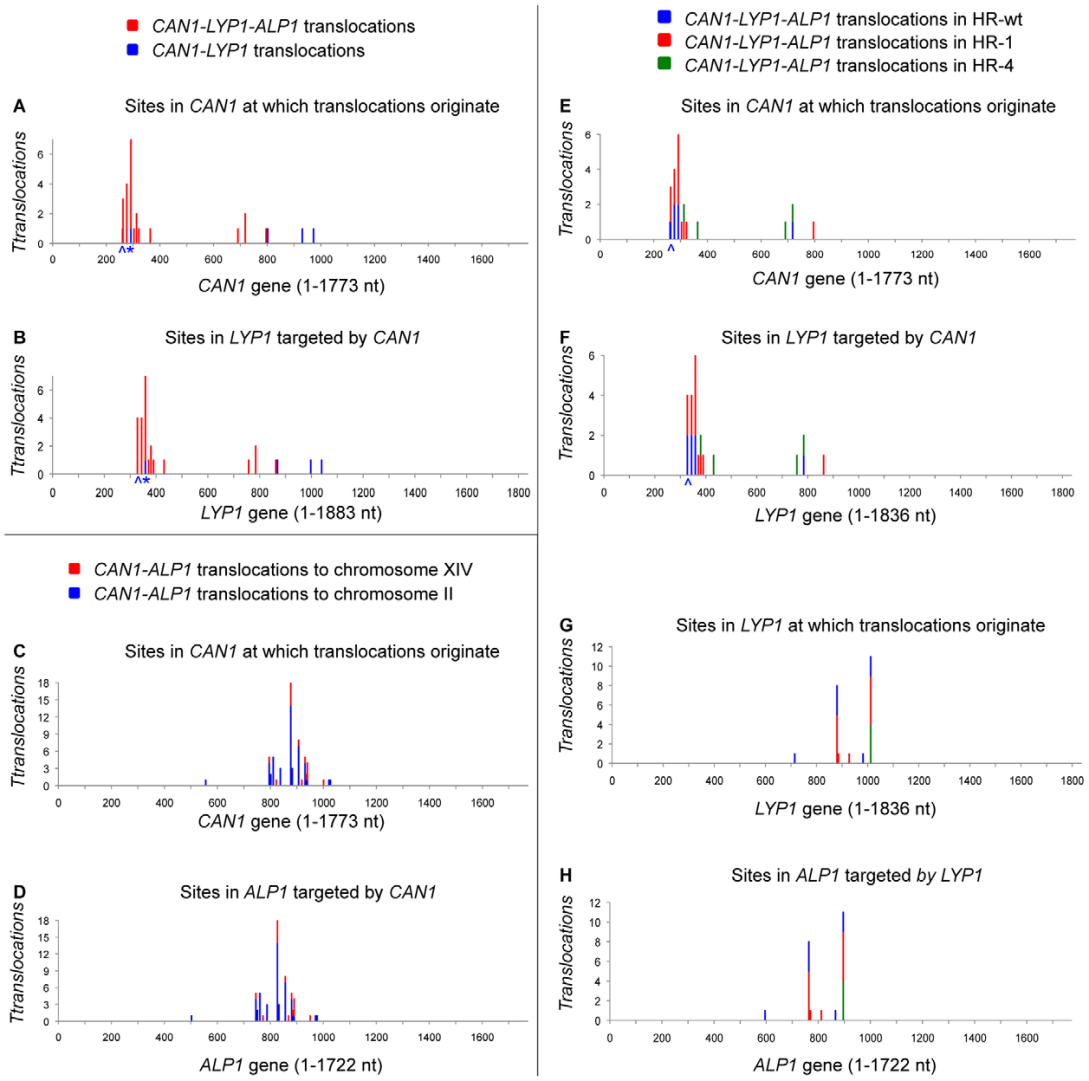
Location of *CAN1* donor sites and *LYP1* and *ALP1* target sites in all translocation types. (**A**) Sites in *CAN1* at which *C/L* translocations originate (blue) and are located downstream of those at which *C/L/A* translocations originate (red). The only exception (labeled *) is a *C/L* translocation in which *ALP1* was not available for an intrachromosomal *C/L/A* rearrangement. Notes that the breakpoint is located within an (AG)_4_ dinucleotide repeat that is susceptible to slippage, resulting in a *CAN1* donor site that does not match the *LYP1* target site (labeled ∧) (**B**) Sites in *LYP1* targeted by *C/L* translocations (blue) are located downstream of those targeted by *C/L/A* translocations (red). (**C–D**) *CAN1* donor sites and *ALP1* target sites of *C/A* translocations to chromosome XIV match *CAN1* donor sites and *ALP1* target sites of C/A translocations to chromosome II originate. (**E–F**) *CAN1* donor and *LYP1* target sites of *C/L/A* translocations in HR-wt, HR-1 and HR-4 fall into the same clusters and match except for the slippage event at the first breakpoint (labeled ∧). (**G–H**) *LYP1* donor sites match *ALP1* target sites. Note that all rearrangements between *LYP1* and *ALP1* in HR-4, in which HER-II sequences match 100%, occur at the same breakpoint (green).

Next we asked why some translocations from *CAN1* to *LYP1* undergo a secondary rearrangement with *ALP1* to form *C/L/A* translocations whereas other translocations from *CAN1* to *LYP1* terminate as *C/L* translocations. We found that the sites in *CAN1* at which *C/L* translocations originate (Figure 3 A, blue) were downstream of sites in *CAN1* at which *C/L/A* translocations originate (Figure 3 A, red), and the *LYP1* target sites in *C/L* translocations were downstream of all *LYP1* target sites in *C/L/A* translocations (Figure 3 B). This finding suggests that sequence similarity between *LYP1* and *ALP1* downstream of these *C/L* breakpoints is insufficient for an rearrangement with *ALP1*. Thus, a translocation from *CAN1* to *LYP1* only results in a viable chromosome if the initiating breakpoint in *CAN1* is located in the HER-I region or at the extreme 5′ end of the HER-II region, so that most of the 96% identical HER-II region is available for a secondary rearrangement between *LYP1* and *ALP1*. Interestingly, we found one translocation that originated in *CAN1* and targeted HER-I of *LYP1* (Figure 3 A, B, labeled *), but failed to go on to become a *C/L/A* translocation, even though the entire HER-II region was available for a secondary rearrangement with *ALP1*. That this *C/L* translocation was identified in the HR-2 strain, in which *LYP1* and *ALP1* were on two different chromosomes, demonstrates that translocations from *CAN1* to *LYP1* get stuck in HER-I when *ALP1* is not available on the same chromosome for a secondary rearrangement. This finding is consistent with our conclusion above that secondary rearrangements between *LYP1* and *ALP1* only occur *intra*chromosomally.

**Figure 4:**
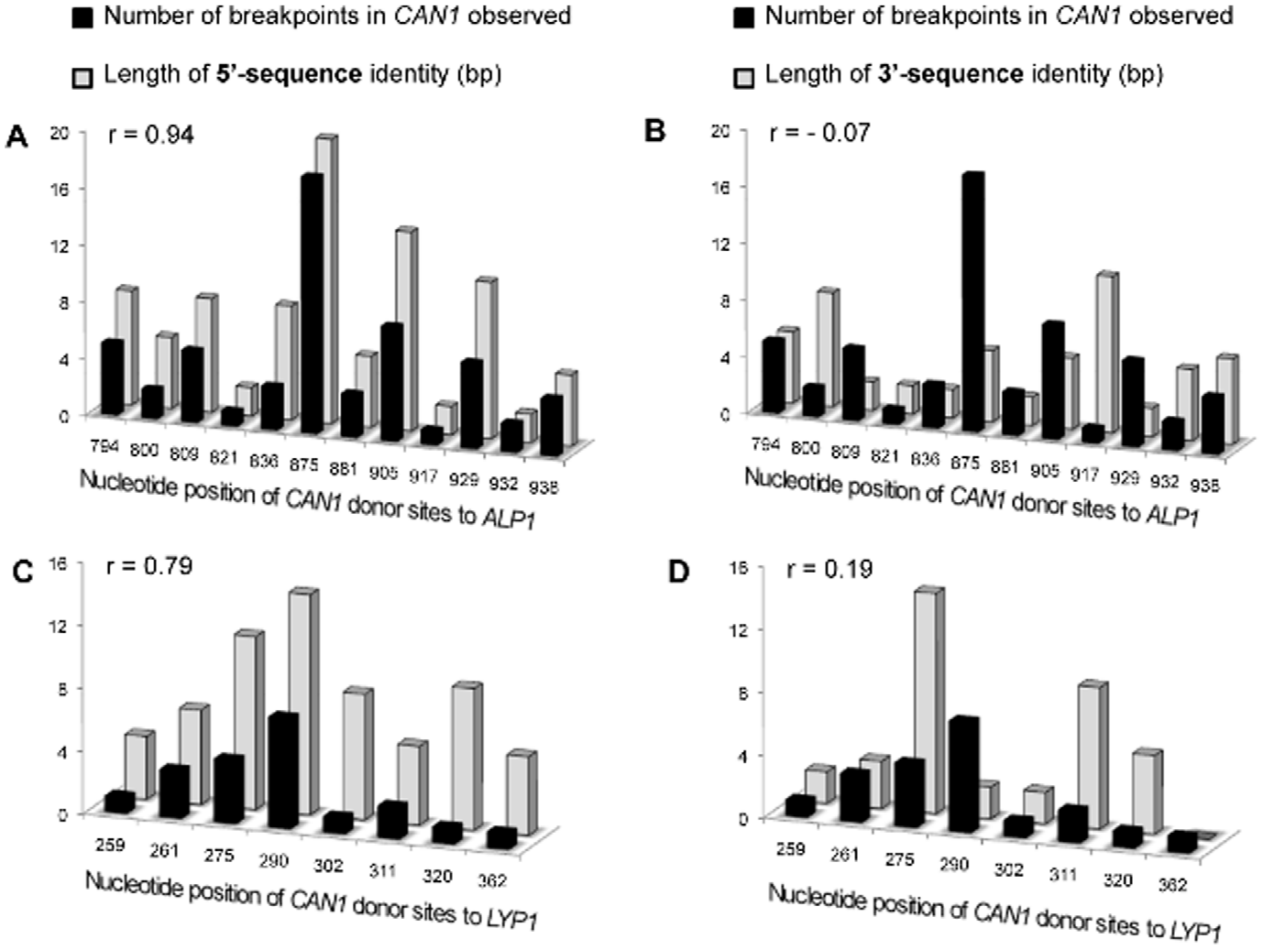
Positive correlation between the number of breakpoints and length of 5′ homology. (**A**) 5′ homology block length in HER-II of *CAN1* shows a positive correlation to the number of *C/A* breakpoints at that site, whereas (**B**) 3′ homology block length shows no correlation. (**C**) 5′ homology block length in HER-I of *CAN1* shows a positive correlation to the number of *C/L/A* translocations originating from that site, whereas (**D**) no correlation is found for 3′ homology block length. The Pearson correlation coefficient (r) is indicated.

To determine how moving *ALP1* to a different chromosome had affected breakpoint selection in *ALP1*, we compared the breakpoint target sites in *ALP1* on chromosome II with those in *ALP1* on chromosome XIV. This analysis revealed that the chromosomal location of *ALP1*, while affecting translocation type, did not influence target site selection within *ALP1* or donor site selection within *CAN1* (Figure 3 C, D). *C/A* translocations, no matter whether they target chromosome XIV (red) or II (blue), originate from nearly identical sets of *CAN1* sites. Similarly, the sites in *ALP1* on chromosome XIV and *ALP1* on chromosome II that are targeted by *CAN1* also match. Thus, we can conclude that while translocation rates are determined by the chromosomal location of the target genes, breakpoint selection is not. This breakpoint analysis also revealed that the location of the breakpoints in *CAN1* exactly predicts the location of the target sites in *ALP1*, as evidenced by the matching patterns of donor sites in *CAN1* and target sites in *ALP1* (Figure 3 C and D). This predictability of breakpoint patterns extends to *C/L/A* translocations (Figure 3 E–H), where donor sites in *CAN1* predict the target sites in *LYP1* (Figure 3 E–F) and donor sites in *LYP1* predetermine the target sites in *ALP1* (Figure 3 G–H). However, no connection appears to exist between *LYP1* sites targeted by *CAN1* and *LYP1* sites serving as a donor for the *L/A* rearrangement. Requirement of the downstream HER-II region in the conversion of dicentric *C/L* translocations to monocentric *C/L/A* translocations indicates 5′ to 3′ directionality of the recombination process. This directionality is further supported by the positive correlation between the length of homology between *CAN1* and *ALP1* as well as between *CAN1* and *LYP1* upstream of the *C/A* and *C/L* breakpoints, respectively, and the number of breakpoints observed at that site, whereas no correlation exists for the downstream sequence (Figure 4). There was only a weak positive correlation between the length of homology between *LYP1* and *ALP1* and the number of *intra*chromosomal *L/A* rearrangements observed at that site (r = 0.38), suggesting that the interchromosomal rearrangement is HR-dependent whereas the intrachromosomal rearrangement may be HR-independent and/or affected by additional constrains.

### Unstable Dicentrics Give Rise to Complex Monocentrics and Intraclonal Heterogeneity

**Figure 5:**
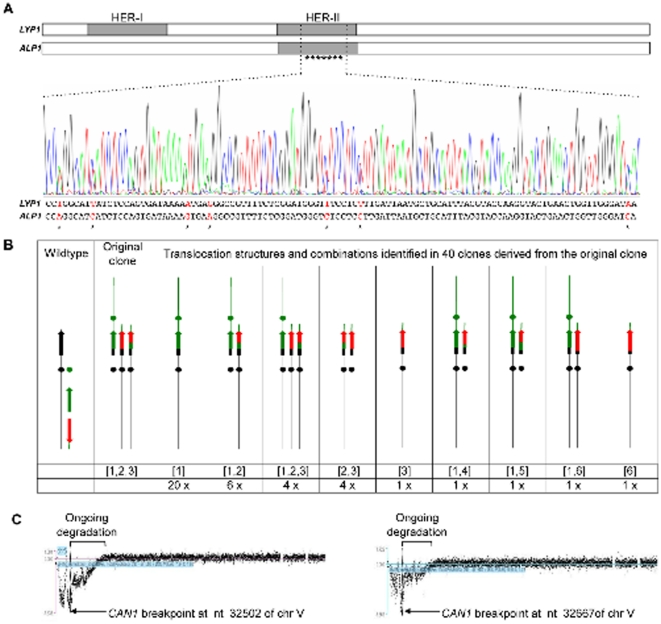
Clonal instability of translocations. (**A**) Double peaks in the chromatogram indicate presence of *C/A* and *C/L/A* translocations in the same clone. Seven single nucleotide differences, indicated in red, distinguish the HER-II regions of *ALP1* and *LYP1*. (**B**) Translocations in forty single colonies derived from unstable clone 1095 were characterized. The locations of *CAN1* (black), *LYP1* (green), *ALP1* (red) in the parental strain are shown on the left; multiple translocations identified in clone 1095 are shown in the column “original clone”; nine different combinations (bottom row) of six different translocations (types 1–6) in the forty single colonies are schematically depicted in the right column. The three translocation types indentified in the original clone are named type 1, type 2 and type 3, indicated below the column as [1], [2], [3]. Three translocations with new breakpoints were observed, named “4”, “5” and “6”. The mixture of various translocations indentified in each clone is indicated in brackets below each column, and the number of clones with a particular translocation mixture is indicated at the bottom of each column. (**C**) aCGH reveals chromosome end degradation in two clones with single *C/L* dicentrics. Clones 608 (top) and 349J (bottom) were isolated from *sgs1Δ mec3Δ* and *sgs1Δ rad24Δ* mutants, respectively. The original *CAN1* breakpoint is indicated by a vertical line.

While *C/L* translocations can be identified by screening with primers that anneal to the 5′ and 3′ ends of *CAN1* and *LYP1*, respectively, primers that anneal to the 5′ and 3′ ends of *CAN1* and *ALP1* amplify both *C/A* and *C/L/A* translocations, which can only be distinguished by sequencing. The simultaneous presence of *C/A* and *C/L/A* translocations in the same clone is indicated by double peaks in the sequencing chromatogram at sites where the homeologous regions in *ALP1* and *LYP1* differ. The identification of such clones (included in ‘Other’ in Table 2) that harbor multiple types of translocation between *CAN1*, *LYP1* and *ALP1* (Figure 5 A) indicates instability of translocation chromosomes. To test this possibility, clone 1095 harboring *C/L*, *C/A* and *C/L/A* translocations was streaked on agar for single colonies with the expectation that we would obtain the three translocations in individual colonies if the translocations were stable. The colonies that were obtained after 3 days of growth were heterogeneous, ranging from tiny to large with round or irregular edges. Of 40 single colonies that were analyzed, 25 contained single translocations that were identical to those found in the original clone. Among the other 15 single colonies, however, three novel translocations with breakpoints not seen in the original clone were identified as well as six new combinations of new and old translocations. Thus, instead of the expected three translocations, a total of six different translocations in nine different combinations were identified (Figure 5 B). Since all 40 colonies were derived from single cells, the identification of single colonies with multiple (old and new) translocations in several combinations indicates that at least one of the original translocations is unstable. The obligatory presence of the original *C/L* translocation in all colonies with multiple new and old translocations suggests that the dicentric *C/L* chromosome is unstable and subjected to cycles of rearrangement that lead to new translocations. In addition to clone 1095, evidence that *C/L* and *C/L/A* translocations present in the same clone may be derived from each other was also found in heterogeneous clone 1063, in which sequencing revealed that all translocations shared the same *CAN1* breakpoint at nucleotide 800 of *CAN1*. Continued instability of chromosome V after having rearranged with *LYP1* to form a dicentric *C/L* translocation is detectable by array-based comparative genome hybridization (array CGH) (Figure 5 C). The two clones with single *C/L* translocations analyzed here were obtained from *sgs1Δ* mutants with defects in the DNA damage checkpoint clamp (*mec3Δ*) or clamp loading (*rad24Δ*), which had previously been shown to yield *C/L* translocations [19]. While CGH on both clones showed that loss of chromosome V sequence is most noticeable distal of the *CAN1* breakpoint (due to loss of this region in the original *C/L* translocation and hence its absence in all its derivatives), it also revealed further degradation beyond the *CAN1* locus, indicating ongoing instability.

### Error-Prone Break-Induced Replication Reveals Common Translocation Origin

**Figure 6:**
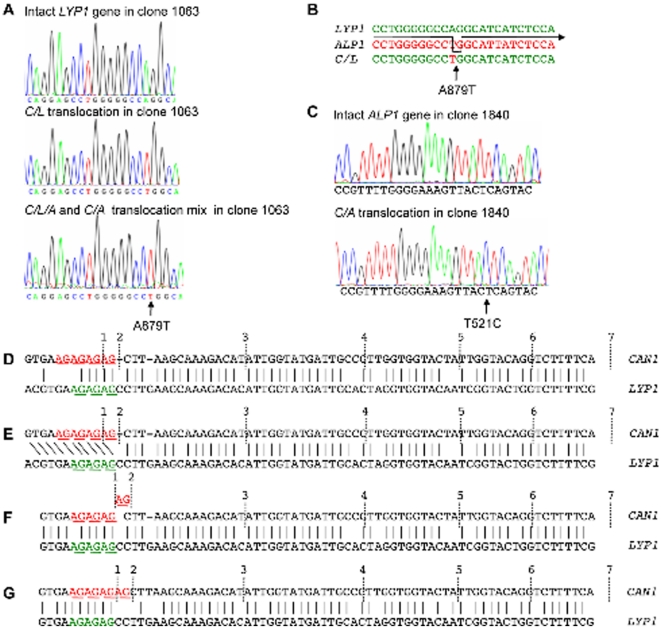
Translocation formation is associated with single nucleotide changes and evidence of DNA slippage. (**A**) An A879T change was observed in the *C/L* and *C/L/A* translocations of clone 1063, but not in *LYP1* of the intact chromosome XIV of that clone, suggesting it arose during translocation formation and *C/L/A* is derived from *C/L*. (**B**) Formation of the A879T change by template switching from *LYP1* to *ALP1* and back to *LYP1*. (**C**) A T521C change in the *C/A* translocation of clone 1840 was not present in *ALP1* on the intact chromosome XIV in the same clone. T521C may have resulted from a replication error or template switching to a locus other than *ALP1*, *LYP1* or *CAN1* since none of them contains a C at this location (**D**) Alignment of HER-I of *CAN1* and *LYP1*, indicating seven breakpoints at which *CAN1* and *LYP1* recombine. Except for breakpoint 1, all are followed by a single mismatch. The (AG)_4_ repeats in *CAN1* and *LYP1* are indicated in red and green, respectively. (**E–G**) Looping out of a single AG unit leads to a longer perfect match between *CAN1* and *LYP1*, and could explain why breakpoints 1 and 2 can be found fused to 5′-CCTT sequence of *LYP1*.

Identical *CAN1* breakpoints shared by multiple translocations in heterogeneous clones were suggestive of a common origin of the various translocations. Thus, we analyzed the DNA sequences downstream of *CAN1* breakpoints in heterogeneous clones for additional shared features. Indeed, in heterogeneous clone 1063 an A879T substitution was identified in the *C/L* translocation 12 nucleotides downstream of the *C/L* breakpoint (Figure 6 A). Since translocations between *CAN1*, *LYP1* and *ALP1* are nonreciprocal, as indicated by the presence of intact *LYP1* and *ALP1* genes on chromosome XIV, analysis of the wildtype *LYP1* gene was possible in this clone. Sequencing revealed that the A879T mutation was not present in the intact *LYP1* gene of this clone, suggesting that the mutation occurred during translocation from *CAN1* to *LYP1*. This A879T substitution could have resulted either from a polymerase error or from *CAN1* invading and copying the nearby *ALP1* locus (which contains a T at this location) prior to forming the *C/L* translocation (Figure 6 B). Upstream of this A/T mismatch *LYP1* and *ALP1* share 41 bp of perfect sequence identity, which could have stabilized such a transient template-switch. If the *C/L/A* translocation in the same clone was indeed derived from this *C/L* translocation, as already suggested by their common *CAN1* breakpoint, the base substitution should also be present. That sequencing of the *C/L/A* translocation indeed identified the same base substitution suggests that these two translocations are derived from each other instead of arising independently from separate *CAN1* invasions. A T521C substitution 21 nucleotides downstream of the *C/A* breakpoint was found in the *C/A* translocation of a another clone (1840), but not in the intact *ALP1* gene of that clone (Figure 6 C). However, since T, not C, is found at the corresponding positions in *CAN1*, *LYP1* and *ALP1*, and a BLAST search of the yeast genome revealed no locus with extensive sequence identity to *ALP1* surrounding the T/C mismatch, the T521C base substitution is likely to be the result of a polymerase error during early BIR.

In addition to base substitutions, we detected possible evidence of DNA slippage (Figure 6 D–G). With a single exception, the *CAN1* sequence in *C/L* and *C/L/A* translocations terminates at single base-pair mismatches within HER-I (Figure 6 D, breakpoints 2–7). Termination at breakpoint 1, which is not followed by a mismatch (Figure 6 D, breakpoint 1) was observed in one *C/L/A* translocation and could be explained by DNA slippage within a short AG repeat during strand invasion. Specifically, slippage of the (AG)_4_ repeat in *CAN1* during annealing to the corresponding (AG)_3_ repeat in *LYP1* may have led to looping out of a single repeat unit in *CAN1* (Figure 6 F) or, more likely, a shift in base pairing (Figure 6 G). As a result, a mismatched base may sometimes be located after (AG)_4_ or after (AG)_3_, thus making both sites susceptible to becoming *CAN1* translocation donor sites. The increase in length of the base-paired region from six to ten matches as a result of this slippage event may have promoted termination of the *CAN1* sequence at breakpoint 1.

## Discussion

Although some RecQ-like helicases have been successfully purified so that their substrate specificity and enzymatic function could be determined *in vitro*, less is known about the mechanism by which RecQ-like DNA helicases preserve genome integrity or about the types of genome rearrangements that arise in cells lacking RecQ-like DNA helicases. In an ongoing effort to shed light on these questions, we previously showed that yeast double mutants lacking the RecQ-like helicase Sgs1 in addition to certain DNA damage checkpoint components (Mec3, Rad24, Tel1) accumulate recurring, Rad52-dependent, Rad51-independent translocations between the related *CAN1*, *LYP1* and *ALP1* genes [19]. Simple *C/A* and *C/L* translocations, but also more complex *C/L/A* rearrangements were found in these mutants, and the aim of the current study was to test models for the formation of these translocations, and to gain additional insight into the general mechanism of translocation formation. One possibility for *C/L/A* translocation formation was that they arise in a single event, in which *CAN1* invades *LYP1*, but dissociates and reanneals to the nearby *ALP1*. Alternatively, *C/L/A* translocations could form as a result of two independent events; in the first event *CAN1* translocates to *LYP1* and forms a dicentric *C/L* chromosome, and in the second event, possibly during anaphase of mitosis, this *C/L* dicentric breaks and invades *ALP1* to form a monocentric *C/L/A* translocation. Interestingly, the inability to form *C/L/A* translocations if all three genes are located on three different chromosomes and the observation of clonal instability point to the following two sources for *C/L/A* translocations. Translocation formation is initiated by a DNA break on chromosome V that leads to invasion of *LYP1* on chromosome XIV, using the sequence homology provided by the HER-I sequence in *CAN1* and *LYP1*. This HER-I-mediated invasion of *LYP1* by *CAN1* leads to initiation of DNA synthesis on chromosome XIV, which may then be subjected to dissociation and re-invasion cycles as previously described [6]. If the re-invading strand mistakenly anneals to the nearby *ALP1*, this time utilizing the 96%-identical HER-II sequence for an *intra*chromosomal template-switching event, a monocentric *C/L/A* translocation forms. If re-invasion occurs at the same site in *LYP1* or BIR simply continues without dissociation, a dicentric *C/L* chromosome forms. This dicentric provides the second source for *C/L/A* translocation formation as it is likely to be susceptible to breakage in mitosis followed by renewed attempts at repair. This ongoing instability of dicentrics is supported by our finding of intraclonal heterogeneity of translocation types. Broken *C/L* dicentrics are likely to utilize *LYP1* sequence contained in them to reinvade chromosome XIV at *LYP1*, forming either a another unstable dicentric or giving rise to a stable, monocentric *C/L/A* chromosome by undergoing an intrachromosomal template-switch to *ALP1*. Repeated cycles of breakage and repair of *C/L* dicentrics are the likely explanation for presence of multiple, different translocations in the same clone and continued formation of new gene fusions in our study.

Thus, while recombination between HER-I sequences of *CAN1* and *LYP1* leads to dicentrics that provide a source for *C/L/A* translocations, it appears that annealing of *CAN1* with HER-II of *LYP1* leads to *C/L* dicentrics that disappear from the population because they are unable to undergo a stabilizing rearrangement with *ALP1* due to lack of downstream homology. In some cases, seemingly stable *C/L* translocations with breakpoints in HER-II could be obtained (Figure 3 A and B). In these cases it is likely that they underwent conversion to a monocentric chromosome, using chromosome XIV sequences other than *ALP1*, without disrupting the *C/L* fusion. In other cases, *C/L* translocations were found to be highly unstable, giving rise to new translocations. Although unstable *C/L* translocations were formed by annealing HER-II of *CAN1* and *LYP1*, the *CAN1* breakpoints were located at the very 5′ end of HER-II, leaving almost all of HER-II available for rearrangements with *ALP1* and formation of stable *C/L/A* translocations. Further evidence that *C/L/A* translocations and can be derived from unstable C/L dicentrics is provided by identical *CAN1* breakpoints and the occurrence of single-nucleotide changes shared by multiple translocations in the same clone. Such base substitutions near translocation breakpoints may result from replication errors or re-invasion at similar sequences and could, combined with the potential for frameshifts due to slippage at the gene fusion site, be a source for mutations and loss of gene function even if recombination occurs between allelic sequences on sister chromatids or homologous chromosomes. These recombination-associated errors were rare, occurring in three of the 573 translocations between *CAN1*, *LYP1* and *ALP1* in HR-wt and HR-1 (0.005%).

Intrachromosomal, faulty template-switching between inverted repeats was recently also proposed in a study by Paek *et al* [35] to account for the formation of dicentric chromosomes in budding yeast, which, similar to the dicentrics in our study, proved to be unstable and substrates for further chromosomal rearrangements. These authors also reported that *intra*chromosomal template-switching is Rad52-independent, which could suggest that the Rad52-dependence of the *CAN1/LYP1/ALP1* translocations studied here is due to the interchromosomal recombination event between *CAN1* and *LYP1* or *CAN1* and *ALP1*, whereas *intra*chromosomal template switching between *LYP1* and *ALP1* may be Rad52-independent. Indeed, the weaker correlation between 5′ homology block length and the number of *L/A* breakpoints observed at that site compared to that for *C/L* or *C/A* breakpoints suggests that additional factors affect template switching and may suggest a lesser role (or no role) for HR in the *intra*chromosomal template-switch between *LYP1* and *ALP1*.

That translocations between *CAN1*, *LYP1* and *ALP1* form so frequently in cells lacking Sgs1 and a DNA damage sensor such as Mec3, but not in the single mutants, most likely stems from the independent roles of these factors in preventing different intermediates of translocation formation, such that in the double mutants increased lesion formation, aberrant lesion processing, greater tolerance for dicentrics and/or the products of their breakage and defective checkpoint activation combine to create conditions suitable for translocation formation. That *C/L* dicentrics are unstable and give rise to multiple new rearrangements suggests that dicentrics break during anaphase to fuse again, entering a cycle of repeated breakage and fusion until a stable translocation chromosome is generated, if ever. This process may be comparable to the futile breakage-fusion-bridge (BFB) cycle observed in multicellular eukaryotes. In cancers where intratumor heterogeneity is common, such as osteosarcoma, a positive correlation has been observed between the number of dicentrics and the frequency of BFBs, which are thought to be a source of mitotic chromosome instability and may in some cases generate complex rearrangements involving multiple chromosomes [36]. Interestingly, increased presence of micronuclei, which are thought to contain chromosome fragments that have resulted from breakage of unresolved BFBs, has been reported for cells from Bloom's syndrome patients and from *BLM* knock-out mice [37], [38]. Indeed, the Hickson laboratory recently showed that BLM localizes to BFBs and to novel ultrafine bridges (UFBs), the latter of which commonly emerge from centromeric regions in normal cells [39], [40], [41]. BFBs and UFBs accumulate in *BLM*-defective cells, and the authors found evidence that BLM is required for efficient and proper resolution of bridge structures, most likely by decatenation, rather than the prevention of bridge formation prior to anaphase [40], [41]. Thus, lack of BLM, or Sgs1 in yeast, may contribute to increased chromosome breakage and occasional large-scale rearrangements and DNA loss. Combining the lack of Sgs1/BLM with dysfunctional Tel1/ATM or Mec3/9-1-1 checkpoint pathways creates conditions under which mitotic chromosome breaks may not be efficiently detected and/or faithfully processed, allowing recurring, complex translocations and unstable dicentrics to arise and persist [19]. DNA lesions that give rise to translocations may be present at increased rates in cells lacking Sgs1, as Sgs1 has been shown to also have roles in the processing of DSBs [26], the resolution of unusual secondary DNA structures, such as G4 tetrads [42], [43], resolution of recombination intermediates [22] and possibly in checkpoint activation itself [44]. Here, we have provided evidence how this increased genome instability can lead to the formation of complex translocations by intragenic, interchromosomal BIR that requires as little as 110 bp of 83% identity with homology blocks that do not exceed 14 bp, and by intrachromosomal template-switching that requires as little as 173 bp of 96% identity separated by 2445 bp. In addition, dicentric chromosomes are a source of intraclonal, and most likely intratumor, heterogeneity, giving rise to not only translocations with new breakpoints, but also cells with new combinations of these chromosome rearrangements. In Bloom's syndrome and other human chromosome instability syndromes such ongoing genome instability is likely to contribute to increased cancer incidence at an earlier age and other characteristic signs of premature aging.

## Supporting Information

Figure S1 Needleman-Wunsch alignments of *CAN1*, *ALP1* and *LYP1*. Alignments of the 5′ends of (A) *CAN1* and *ALP1* and (B) the 5′ends of *CAN1* and *LYP1* reveal greater sequence similarity and longer continuous regions of identical sequences between *CAN1* and *LYP1* than *CAN1* and *ALP1*.
(1.19 MB TIF)
