# Supplementary figures and images for: Formation of Complex and Unstable Chromosomal Translocations in Yeast
Source: PLoS One. 2010 Aug 9;5(8):e12007. doi: 10.1371/journal.pone.0012007 (PMC2918500; doi:10.1371/journal.pone.0012007)

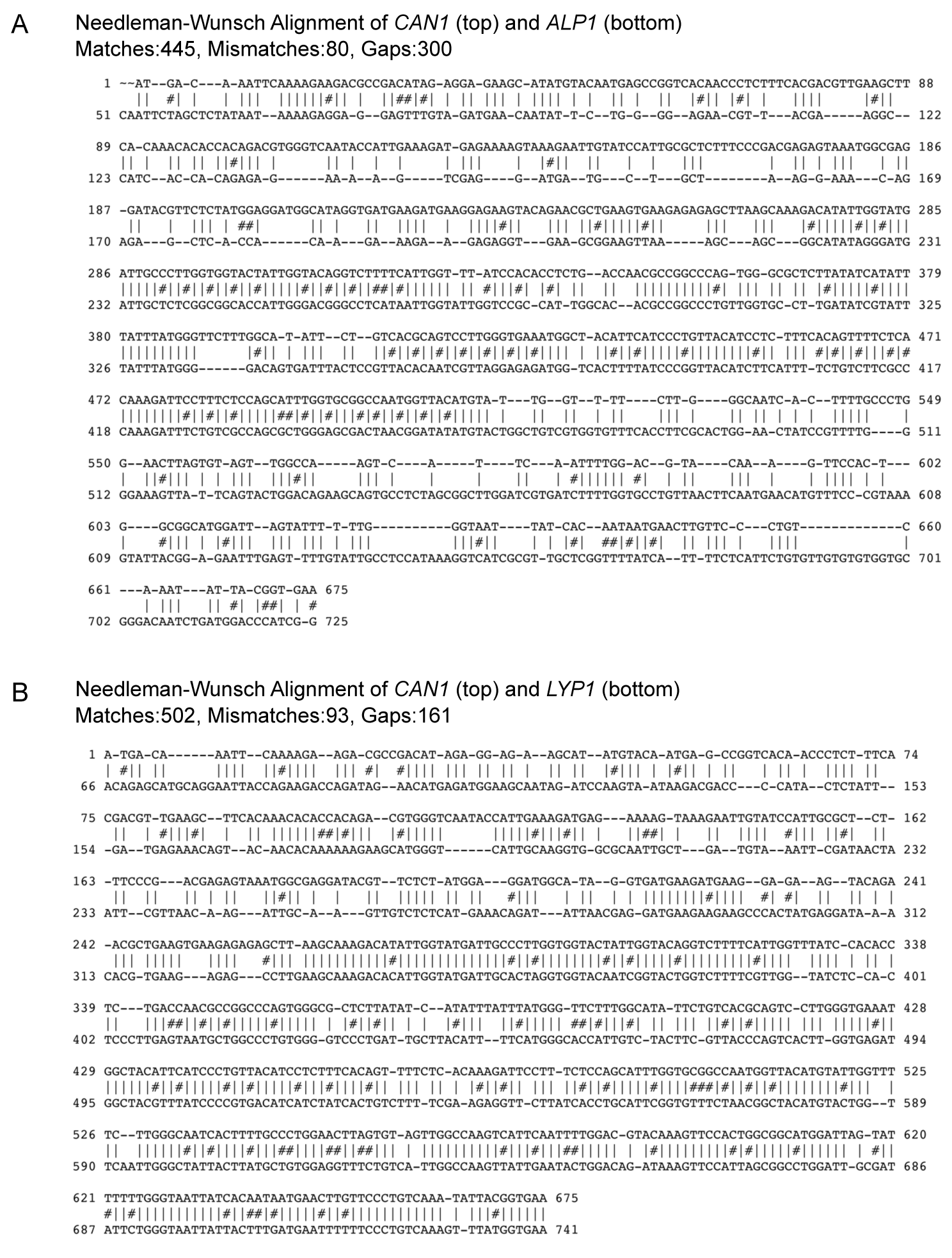

Supplement: Figure S1 — Needleman-Wunsch alignments of CAN1, ALP1 and LYP1. Alignments of the 5′ends of (A) CAN1 and ALP1 and (B) the 5′ends of CAN1 and LYP1 reveal greater sequence similarity and longer continuous regions of identical sequences between CAN1 and LYP1 than CAN1 and ALP1. (1.19 MB TIF) [file pone.0012007.s001.tif]
